# Reply to: The PulMiCC Trial Provides Control Data for Colorectal Lung Metastases Amenable to Local Treatments

**DOI:** 10.1007/s00270-020-02740-x

**Published:** 2021-01-26

**Authors:** Bobby Bhartia, Jim Zhong, Nilanjan Chaudhuri, Richard Milton, Jonathan Smith, James Lenton, Tze Min Wah

**Affiliations:** 1 Clinical Lead for Interventional Oncology, Division of Diagnostic and Interventional Radiology, Leeds Cancer Centre, Institute of Oncology, St. James’s University Hospital, Beckett Street, Leeds, LS9 7TF United Kingdom; 2 Division of Thoracic Surgery, Leeds Cancer Centre, Institute of Oncology, St. James’s University Hospital, Beckett Street, Leeds, LS9 7TF United Kingdom

Dear Editor,

We thank William et al. letter to Editor [1] for their response to the study “Long-Term Outcomes in Percutaneous Radiofrequency Ablation for Histologically Proven Colorectal Lung Metastasis” [2] and for sharing the updated findings of their multi-centre randomised control trial (RCT), which compares active monitoring with lung metastasectomy [3]. The updated PulMiCC trial analysis was published online on 9th May 2020, which was just after our manuscript submission on 5th May 2020. The purpose of our study was to demonstrate the long-term technical outcomes of radiofrequency ablation in histologically proven metastatic disease. Our results were consistent with published series without histological confirmation and also with surgical series. Comparisons, favourable or otherwise, were made with other surgical and ablative modalities rather than with observation. Our reference to the PulMiCC trial was in the context of whether there was any value in further research directly comparing surgery and ablation and intended only to illustrate the challenges faced when conducting RCT in this field. The original PulMiCC study concluded “Because of poor and worsening recruitment, the study was stopped”[4]. We recognise the uncertainties around a survival benefit, the limitations of case series due to selection bias, and the challenges of achieving a RCT. Contrary to the statement that “Those who do not welcome the results of PulMiCC are very ready to dismiss it without further consideration as was done by Zhong et al.”, the authors greatly welcome the publication. NICE guidance, which referenced the original PulMiCC trial analysis found the evidence inconclusive [5]. We acknowledge the importance of the updated PulMiCC trial analysis (*n* = 93) of a better than expected survival in those who do not undergo treatment and the questions this raises regarding the benefit of interventions for colorectal lung metastases. Our patient is always in the heart of our multidisciplinary cancer treatment discussion, and we will always endeavour to provide our patients with all the evidence-based treatment options such as observation and active intervention, e.g. surgery versus image-guided ablative therapy. We hope there will be more published level 1 evidence in the future to inform us regarding the various management options for our patients with colorectal metastasis.
